# Annual change in the extracellular fluid/intracellular fluid ratio and mortality in patients undergoing maintenance hemodialysis

**DOI:** 10.1038/s41598-021-04366-6

**Published:** 2022-01-07

**Authors:** Takahiro Yajima, Kumiko Yajima, Hiroshi Takahashi

**Affiliations:** 1 Department of Nephrology, Matsunami General Hospital, Gifu, 501-6062 Japan; 2 Department of Internal Medicine, Matsunami General Hospital, Gifu, 501-6062 Japan; 3 Division of Medical Statistics, Fujita Health University School of Medicine, Aichi, 470-1192 Japan

**Keywords:** Medical research, Nephrology

## Abstract

We aimed to investigate whether annual change in the extracellular fluid to intracellular fluid (ΔECF/ICF) ratio can accurately predict mortality in hemodialysis patients. Totally, 247 hemodialysis patients were divided into two groups according to the median baseline ECF/ICF ratio of 0.563 and ΔECF/ICF ≥ 0% or < 0% during the first year, respectively. Thereafter, they were divided into four groups according to each cutoff point and were followed up for mortality assessment. The ECF/ICF ratio increased from 0.566 ± 0.177 to 0.595 ± 0.202 in the first year (*P* = 0.0016). During the 3.4-year median follow-up, 93 patients died (42 cardiovascular-specific causes). The baseline ECF/ICF ≥ 0.563 and ΔECF/ICF ≥ 0% were independently associated with all-cause mortality (adjusted hazard ratio [aHR] 4.55, 95% confidence interval [CI] 2.60–7.98 and aHR 8.11, 95% CI 3.47–18.96, respectively). The aHR for ECF/ICF ≥ 0.563 and ΔECF/ICF ≥ 0% vs. ECF/ICF < 0.563 and ΔECF/ICF < 0% was 73.49 (95% CI 9.45–571.69). For model discrimination, adding the ΔECF/ICF (0.859) alone and both the baseline ECF/ICF and ΔECF/ICF (0.903) to the established risk model (0.746) significantly improved the C-index. Similar results were obtained for cardiovascular mortality. In conclusion, the ΔECF/ICF ratio could not only predict all-cause and cardiovascular mortality but also improve predictability of mortality in hemodialysis patients.

## Introduction

Protein-energy wasting (PEW), a malnutritional status defined as loss of body proteins and fuel reserves due to chronic inflammation, is prevalent and associated with morbidity and mortality in patients undergoing hemodialysis (HD)^1–3^. We have recently proposed that bioelectrical impedance analysis (BIA)-measured extracellular fluid to intracellular fluid (ECF/ICF) ratio, which simultaneously reflects ECF volume and body cell mass, may be a novel marker of PEW in patients undergoing maintenance HD^4^. The ECF/ICF ratio can also be used as a promising predictor of cardiovascular events and all-cause and cardiovascular mortality in this population^4,5^.

However, the nutritional status may change constantly; therefore, a regular nutritional assessment may be recommended^6^. We have recently reported that the geriatric nutritional risk index (GNRI), a marker of PEW, significantly decreased during the first follow-up year, and its annual change improved the predictability of all-cause and cardiovascular mortality in patients undergoing maintenance HD^7^. Thus, we hypothesized that the ECF/ICF ratio might also constantly change during the follow-up period, and an increase in the ECF/ICF ratio may be associated with an increased risk of mortality. However, the association between change in the ECF/ICF ratio and mortality remains unknown in this population.

The present study aimed to examine the relationship between the annual change in the ECF/ICF ratio (ΔECF/ICF ratio) with all-cause and cardiovascular mortality in patients undergoing HD. Moreover, we also investigated whether the ΔECF/ICF ratio could improve the predictive accuracy for mortality when it was added to the established risk factors.

## Results

### Baseline characteristics

A total of 247 patients (age, 63.7 ± 13.9 years; male, 67.6%; HD vintage, 0.86 [0.55–4.78] years) were included at baseline, and the characteristics of the present study are summarized in Table 1. A history of CVD was seen in 62.8% of the patients. Serum creatinine, high-density lipoprotein (HDL) cholesterol, phosphorus, and C-reactive protein levels were 8.8 ± 2.9 mg/dL, 44 ± 15 mg/dL, 5.1 ± 1.3 mg/dL, and 0.15 (0.06–0.38) mg/dL, respectively. The cardiothoracic ratio (CTR) was 49.4 ± 5.2%. The baseline GNRI was 93.2 ± 6.6. The mean and median ECF/ICF ratios were 0.60 ± 0.21 and 0.563 (0.455–0.714), respectively.

**Table 1:** Baseline characteristics of the study participants.

	Baseline (*N* = 247)	The four groups (*N* = 198)				*P *value
	All patients	G1 (*N* = 44)	G2 (*N* = 36)	G3 (*N* = 66)	G4 (*N* = 52)	
Age (years)	63.7 ± 13.9	57.0 ± 13.9	67.9 ± 10.7	59.9 ± 14.7	68.7 ± 10.4	< 0.0001
Male (%)	67.6	59.1	77.8	62.1	76.9	0.10
Underlying kidney disease						0.030
Diabetic kidney disease (%)	43.7	40.9	41.7	42.4	50.0	
Chronic glomerulonephritis (%)	28.3	38.6	13.9	39.4	25.0	
Nephrosclerosis (%)	19.0	9.1	36.1	15.2	17.3	
Others (%)	9.0	11.4	8.3	3.0	7.7	
HD duration (years)	0.86 (0.55–4.78)	0.69 (0.55–2.95)	0.60 (0.54–2.42)	1.51 (0.57–6.33)	1.94 (0.61–8.27)	0.0079
Alcohol (%)	22.2	15.9	27.8	19.7	25.0	0.54
Smoking (%)	25.9	34.1	30.6	24.2	23.1	0.57
Hypertension (%)	95.1	97.7	94.4	93.9	98.1	0.60
Diabetes (%)	53.8	45.5	47.2	42.4	53.8	0.66
History of CVD (%)	62.8	50.0	63.9	62.1	78.8	0.032
Dw (kg)	57.3 ± 12.4	59.6 ± 13.4	58.5 ± 9.0	58.5 ± 13.6	55.9 ± 11.1	0.47
BMI (kg/m^2^)	22.1 ± 3.9	22.6 ± 4.0	21.8 ± 2.6	22.7 ± 4.1	21.6 ± 3.8	0.39
CTR (%)	49.4 ± 5.2	47.6 ± 4.0	49.1 ± 4.8	49.0 ± 5.1	50.3 ± 4.7	0.056
BUN (mg/dL)	59.2 ± 15.7	61.0 ± 14.7	57.8 ± 13.0	66.4 ± 16.9	54.6 ± 13.8	0.0003
Creatinine (mg/dL)	8.8 ± 2.9	9.4 ± 2.7	7.8 ± 2.4	9.9 ± 3.6	8.3 ± 2.4	0.0013
Albumin (g/dL)	3.6 ± 0.4	3.8 ± 0.3	3.5 ± 0.3	3.8 ± 0.3	3.6 ± 0.3	< 0.0001
Hemoglobin (g/dL)	10.8 ± 1.3	10.8 ± 1.1	10.4 ± 1.4	11.0 ± 1.3	10.7 ± 1.3	0.075
T-Cho (mg/dL)	154 ± 36	161 ± 36	146 ± 36	160 ± 36	148 ± 36	0.097
HDL-C (mg/dL)	44 ± 15	46 ± 15	43 ± 12	46 ± 16	41 ± 11	0.10
TG (mg/dL)	119 ± 78	115 ± 69	106 ± 55	150 ± 109	103 ± 48	0.0050
Uric acid (mg/dL)	6.9 ± 1.7	7.6 ± 1.3	6.2 ± 1.9	7.4 ± 1.9	6.5 ± 1.6	0.0003
Calcium (mg/dL)	8.9 ± 0.8	8.7 ± 0.8	8.7 ± 0.8	9.1 ± 0.7	9.1 ± 0.9	0.021
Phosphorus (mg/dL)	5.1 ± 1.3	5.2 ± 1.2	5.0 ± 1.3	5.5 ± 1.4	4.7 ± 1.1	0.018
iPTH (pg/mL)	123 (55–223)	140 (73–255)	145 (71–232)	114 (53–207)	100 (16–230)	0.45
Glucose (mg/dL)	140 ± 61	137 ± 58	135 ± 55	136 ± 65	147 ± 63	0.73
CRP (mg/dL)	0.15 (0.06–0.38)	0.10 (0.05–0.23)	0.21 (0.06–0.34)	0.12 (0.05–0.25)	0.15 (0.05–0.53)	0.27
GNRI	93.2 ± 6.6	96.1 ± 6.4	92.0 ± 4.8	95.6 ± 5.0	92.4 ± 5.4	0.0001
ECF/TBW ratio at baseline	0.37 ± 0.08	0.31 ± 0.04	0.42 ± 0.05	0.31 ± 0.05	0.42 ± 0.05	< 0.0001
ICF/TBW ratio at baseline	0.63 ± 0.08	0.69 ± 0.04	0.58 ± 0.05	0.69 ± 0.05	0.58 ± 0.05	< 0.0001
ECF/TBW ratio at one year later	NA	0.29 ± 0.05	0.39 ± 0.05	0.34 ± 0.05	0.42 ± 0.06	< 0.0001
ICF/TBW ratio at one year later	NA	0.71 ± 0.05	0.61 ± 0.05	0.66 ± 0.05	0.58 ± 0.06	< 0.0001
ΔECF/TBW ratio	NA	− 5.8 (− 12.6 to − 0.5)	− 7.7 (− 14.8 to − 1.1)	12.7 (7.1–19.6)	1.1 (− 6.0 to 9.1)	< 0.0001
ΔICF/TBW ratio	NA	2.6 (3.4–5.0)	5.1 (1.5–10.5)	− 5.1 (− 8.5 to − 3.2)	− 1.2 (− 5.7 to 4.3)	< 0.0001
ECF/ICF ratio at baseline	0.60 ± 0.21	0.46 ± 0.08	0.75 ± 0.15	0.44 ± 0.09	0.70 ± 0.13	< 0.0001
ECF/ICF ratio at one year later	NA	0.40 ± 0.09	0.63 ± 0.15	0.54 ± 0.12	0.81 ± 0.18	< 0.0001
ΔECF/ICF ratio (%)	NA	− 9.5 (− 17.3 to − 3.8)	− 14.6 (− 23.0 to − 6.5)	20.9 (10.5 to 31.4)	15.4 (6.3 to 22.9)	< 0.0001

BMI, body mass index; BUN, blood urea nitrogen; CRP, C-reactive protein; CTR, cardiothoracic ratio; CVD, cardiovascular disease; Dw, dry weight; ECF, extracellular fluid; ICF, intracellular fluid; TBW, total body water; ECF/ICF ratio, extracellular fluid/intracellular fluid ratio; ΔECF/ICF ratio, annual change in the ECF/ICF ratio; GNRI, geriatric nutritional risk index; HD, hemodialysis; HDL-C, high-density lipoprotein cholesterol; iPTH, intact parathyroid hormone; NA, not available; T-Cho, total cholesterol; TG, triglycerides. G1, ECF/ICF ratio < 0.563, and ΔECF/ICF ratio < 0%; G2, ECF/ICF ratio ≥ 0.563 and ΔECF/ICF ratio < 0%; G3, ECF/ICF ratio < 0.563 and ΔECF/ICF ratio ≥ 0%; G4, ECF/ICF ≥ 0.563 and ΔECF/ICF ≥ 0%.

### The associations of the baseline ECF/ICF ratio with all-cause and CVD mortality

During the median follow-up of 3.4 (1.9–5.6) years, 93 patients died due to CVD (*N* = 42, 45.2%), infection (*N* = 27, 29.0%), cancer (*N* = 13, 14.0%), and other causes (*N* = 11, 11.8%).

In the multivariate Cox proportional hazard analysis, the baseline ECF/ICF ratio was a significant predictor of all-cause mortality (adjusted hazard ratio [aHR], 10.96; 95% confidence interval [CI], 3.41–35.19; P < 0.0001) even after adjusting for sex and age, serum creatinine, HDL cholesterol, phosphorus, C-reactive protein, history of CVD, CTR, and GNRI, which were significant covariates in the univariate analysis. When the patients were divided according to the median baseline ECF/ICF ratio of 0.563 into lower and higher groups (baseline ECF/ICF ratio < 0.563 vs. baseline ECF/ICF ratio ≥ 0.563), the 11-year all-cause survival rates in the lower and higher groups were 66.8% and 11.6%, respectively (P < 0.0001). A higher baseline ECF/ICF ratio was independently associated with an increased risk of all-cause mortality (aHR, 4.24; 95% CI 2.37–7.61; P < 0.0001). Similar results were obtained for cardiovascular mortality.

### The associations of the ΔECF/ICF ratio and baseline ECF/ICF ratio with all-cause and CVD mortality

**Figure 1:**
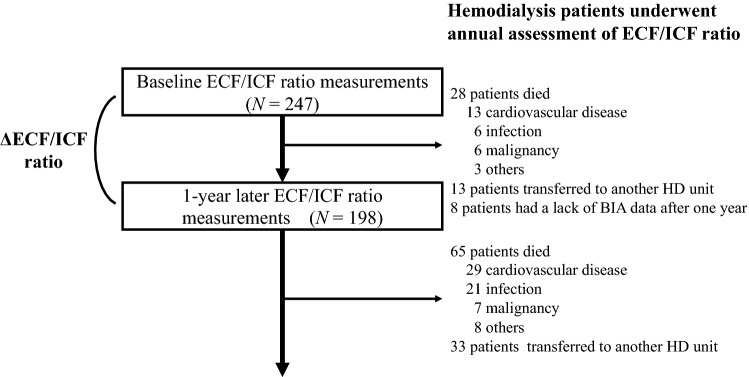
Flow diagram of the present study. ECF/ICF ratio, extracellular fluid/intracellular fluid ratio; HD, hemodialysis.

**Figure 2:**
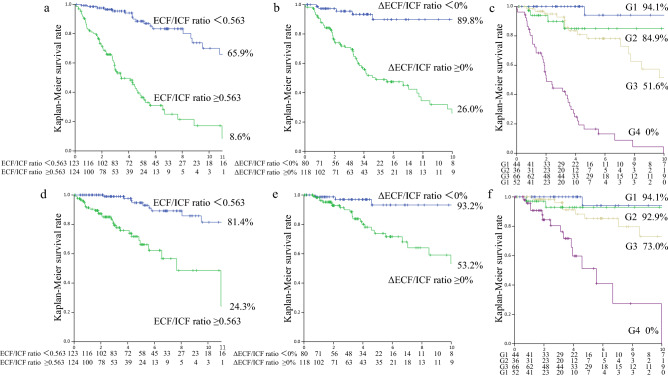
Kaplan–Meier survival rate for all-cause and cardiovascular mortality. All-cause mortality for the ECF/ICF ratio < 0.563 vs. the ECF/ICF ratio ≥ 0.563 (**a**), ∆ECF/ICF ratio < 0% vs. ∆ECF/ICF ratio ≥ 0% (**b**), and among the four groups divided by the baseline ECF/ICF ratio and ∆ECF/ICF ratio (**c**). Cardiovascular mortality for the ECF/ICF ratio < 0.563 vs. the ECF/ICF ratio ≥ 0.563 (**d**), ∆ECF/ICF ratio < 0% vs. ∆ECF/ICF ratio ≥ 0% (**e**), and among the four groups divided by the ECF/ICF ratio and the ∆ECF/ICF ratio (**f**). G1, ECF/ICF ratio < 0.563 and ΔECF/ICF ratio < 0%; G2, ECF/ICF ratio ≥ 0.563 and ΔECF/ICF ratio < 0%; G3, ECF/ICF ratio < 0.563 and ΔECF/ICF ratio ≥ 0%; G4, ECF/ICF ≥ 0.563 and ΔECF/ICF ≥ 0%. ECF/ICF ratio, extracellular fluid/intracellular fluid ratio; ΔECF/ICF ratio, annual change in ECF/ICF ratio.

During the first year, 28 patients died (CVD, 13; infection, 6; cancer, 6; other causes, 3), 13 patients transferred to another HD unit, and 8 patients lacked BIA data after 1 year (Fig. 1). These 49 patients were excluded, and the remaining 198 patients were analyzed to evaluate the associations of the ΔECF/ICF ratio and baseline ECF/ICF ratio with all-cause and CVD mortality (Fig. 1). The ECF/ICF ratio significantly increased from 0.566 ± 0.177 to 0.595 ± 0.202 (*P* = 0.0016) during the first year. The ΔECF/ICF ratio was significantly correlated with the baseline ECF/ICF ratio (ρ = − 0.187, *P* = 0.0083). In the multivariate Cox proportional hazards analysis, the ∆ECF/ICF ratio was an independent predictor of all-cause mortality (aHR, 1.02; 95% CI 1.01–1.03; *P* = 0.0002) (Table 2). When patients were divided by the decreased or increased ∆ECF/ICF ratio in the first year (∆ECF/ICF < 0% vs. ∆ECF/ICF ≥ 0%), the 10-year all-cause survival rates were 89.8% and 26.0%, respectively (P < 0.0001) (Fig. 1). The increased ∆ECF/ICF ratio was an independent predictor of all-cause mortality (aHR, 8.22; 95% CI 3.44–7.61; P < 0.0001) (Table 2). Moreover, when patients were divided by the median of the baseline ECF/ICF ratio and the increased or decreased ∆ECF/ICF ratio into G1, G2, G3, and G4 groups, the 10-year all-cause survival rates were 94.1%, 84.9%, 51.6%, and 0% in G1, G2, G3, and G4, respectively (P < 0.0001) (Fig. 2). The all-cause death number, observational period, and all-cause crude mortality rate were also shown in Table 3. The aHRs for all-cause mortality were as follows: 5.43 (95% CI 0.59–50.29, *P* = 0.14) for G2 vs. G1, 11.52 (95% CI 1.51–87.98, *P* = 0.019) for G3 vs. G1, and 73.49 (95% CI 9.45–571.69, P < 0.0001) for G4 vs. G1 (Table 2). Similar results were obtained for cardiovascular mortality (Fig. 2, Table 2).

**Table 2:** Cox proportional hazards analysis of the ECF/ICF ratio and the ΔECF/ICF ratio for all-cause and cardiovascular mortality.

Variables	Univariate		Multivariate	
	HR (95% CI)	*P* value	HR (95% CI)	*P* value
**All-cause mortality**				
ECF/TBW ratio*	1.14 (1.11–1.17)	< 0.0001	1.10 (1.06–1.14)	< 0.0001
ΔECF/TBW ratio	0.99 (0.98–1.01)	0.33	1.00 (0.99–1.02)	0.76
ICF/TBW ratio*	0.88 (0.85–0.90)	< 0.0001	0.91 (0.88–0.94)	< 0.0001
ΔICF/TBW ratio	1.01 (0.98–1.04)	0.37	0.99 (0.97–1.02)	0.76
ECF/ICF ratio (continuous)	47.04 (20.19–107.18)	< 0.0001	10.96 (3.41–35.19)	< 0.0001
ΔECF/ICF ratio (continuous)	1.02 (1.01–1.03)	0.0005	1.02 (1.01–1.03)	0.0002
ECF/ICF ratio ≥ 0.563	7.17 (4.27–12.03)	< 0.0001	4.24 (2.37–7.61)	< 0.0001
ΔECF/ICF ratio ≥ 0%	7.31 (3.16–16.94)	< 0.0001	8.22 (3.44–19.65)	< 0.0001
Cross-classified (vs. G1)		< 0.0001		< 0.0001
G2	10.29 (1.19–88.71)	0.034	5.43 (0.59–50.29)	0.14
G3	11.56 (1.54–86.93)	0.017	11.52 (1.51–87.98)	0.019
G4	86.67 (11.72–640.80)	< 0.0001	73.49 (9.45–571.69)	< 0.0001
**Cardiovascular mortality**				
ECF/TBW ratio*	1.13 (1.09–1.18)	< 0.0001	1.07 (1.02–1.13)	0.0062
ΔECF/TBW ratio	0.99 (0.97–1.01)	0.46	1.00 (0.98–1.03)	0.90
ICF/TBW ratio*	0.88 (0.85–0.92)	< 0.0001	0.93 (0.88–0.98)	0.0070
ΔICF/TBW ratio	1.01 (0.97–1.06)	0.56	0.99 (0.95–1.04)	0.81
ECF/ICF ratio (continuous)	46.72 (12.54–165.11)	< 0.0001	6.62 (1.22–35.79)	0.028
ΔECF/ICF ratio (continuous)	1.02 (1.01–1.03)	0.0047	1.03 (1.01–1.05)	0.0009
ECF/ICF ratio ≥ 0.563	6.50 (3.12–13.56)	< 0.0001	3.69 (1.56–8.71)	0.0029
ΔECF/ICF ratio ≥ 0%	4.71 (1.64–13.55)	0.0040	6.11 (2.01–18.65)	0.0015
Cross-classified (vs. G1)		< 0.0001		0.0001
G2	7.25 (0.74–70.88)	0.088	5.13 (0.42–63.30)	0.20
G3	6.84 (0.87–53.45)	0.067	9.56 (1.11–81.84)	0.039
G4	40.36 (5.10–319.50)	0.0005	44.72 (4.54–440.47)	0.0011

*Per 0.01 increased. ECF, extracellular fluid; ICF, intracellular fluid; TBW, total body water; ΔECF/TBW ratio, annual change in ECF/TBW ratio; ΔICF/TBW ratio, annual change in ICF/TBW ratio; ΔECF/ICF ratio, annual change in ECF/ICF ratio. G1, ECF/ICF ratio < 0.563 and ΔECF/ICF ratio < 0%; G2, ECF/ICF ratio ≥ 0.563 and ΔECF/ICF ratio < 0%; G3, ECF/ICF ratio < 0.563 and ΔECF/ICF ratio ≥ 0%; G4, ECF/ICF ≥ 0.563 and ΔECF/ICF ≥ 0%. All-cause mortality: adjusted by age, sex, history of cardiovascular disease, creatinine, phosphorus, C-reactive protein, high-density lipoprotein cholesterol, cardiothoracic ratio, and the geriatric nutritional risk index. Cardiovascular mortality: adjusted by age, sex, history of cardiovascular disease, phosphorus, cardiothoracic ratio, and the geriatric nutritional risk index.

**Table 3:** All-cause and cardiovascular death number, observation period, and crude mortality rate in the divided four groups.

	All-cause death number (cardiovascular)	Observational period (years)	All-cause crude mortality rate (%) (cardiovascular)
G1 (N = 44)	1 (1)	5.8 ± 3.5	2.3 (2.3)
G2 (N = 36)	5 (3)	4.4 ± 2.5	13.9 (8.3)
G3 (N = 66)	17 (10)	6.0 ± 3.5	25.8 (15.2)
G4 (N = 52)	42 (15)	3.6 ± 2.0	80.8 (28.8)

### Model discrimination

In the model discrimination of all-cause mortality, the C-index tended to increase from 0.746 to 0.795 (*P* = 0.071) when the baseline ECF/ICF ratio was added to the established risk model including sex and age, serum creatinine, HDL cholesterol, phosphorus, C-reactive protein, history of CVD, CTR, and GNRI (Table 4). The C-index significantly increased when ∆ECF/ICF alone (0.859, *P* = 0.0002) and both baseline ECF/ICF and ∆ECF/ICF (0.903, P < 0.0001) were added to the established risk model, respectively (Table 4). Furthermore, the net reclassification improvement (NRI) and integrated discrimination improvement (IDI) for all-cause mortality significantly improved by adding the ECF/ICF ratio alone, ∆ECF/ICF ratio alone, and both baseline ECF/ICF ratio and ∆ECF/ICF ratio to the established risk model, respectively. For cardiovascular mortality, the addition of the baseline ECF/ICF ratio and ∆ECF/ICF ratio to the established risk model tended to improve the C-index from 0.714 to 0.774 (*P* = 0.076) and significantly improved the NRI (0.624, *P* = 0.0009) and IDI (0.047, *P* = 0.0005) (Table 4).

**Table 4:** Predictive accuracy of the ECF/ICF ratio and/or the ΔECF/ICF ratio for all-cause and cardiovascular mortality.

Variables	C-index	*P* value	NRI	*P* value	IDI	*P* value
**All-cause mortality**						
Established risk factors*	0.746 (0.671–0.821)		Ref		Ref	
+ ECF/ICF ratio	0.795 (0.727–0.862)	0.071	0.724	< 0.0001	0.069	0.0003
+ ΔECF/ICF ratio	0.859 (0.806–0.913)	0.0002	0.943	< 0.0001	0.188	< 0.0001
+ ECF/ICF ratio and ΔECF/ICF ratio	0.903 (0.859–0.948)	< 0.0001	0.919	< 0.0001	0.307	< 0.0001
**Cardiovascular mortality**						
Established risk factors**	0.714 (0.609–0.819)		Ref		Ref	
+ ECF/ICF ratio	0.713 (0.606–0.821)	0.97	0.285	0.078	0.009	0.13
+ ΔECF/ICF ratio	0.764 (0.671–0.858)	0.13	0.466	0.010	0.061	0.0003
+ ECF/ICF ratio and ΔECF/ICF ratio	0.774 (0.684–0.865)	0.076	0.624	0.0009	0.047	0.0005

ECF/ICF ratio, extracellular fluid/intracellular fluid ratio; ΔECF/ICF ratio, annual change in ECF/ICF ratio.

*Age, sex, history of cardiovascular disease, creatinine, phosphorus, C-reactive protein, high-density lipoprotein cholesterol, cardiothoracic ratio, and the geriatric nutritional risk index.

**Age, sex, history of cardiovascular disease, phosphorus, cardiothoracic ratio, and the geriatric nutritional risk index.

## Discussion

The present study demonstrated that the ECF/ICF ratio increased in the first year and that an increased ∆ECF/ICF ratio was independently associated with an increased risk of mortality in patients undergoing HD. Moreover, the predictability for mortality significantly improved when the ∆ECF/ICF ratio alone or both baseline ECF/ICF ratio and ∆ECF/ICF ratio were added to the established risk model. Therefore, our findings suggest that repeated measurements of the ECF/ICF ratio may be useful to precisely predict mortality in this population.

The ECF/ICF ratio measured by BIA has emerged as an indicator that simultaneously reflects the ECF volume and nutritional status in HD patients, with an increase in ECF indicating an excess of ECF and a decrease in ICF indicating a reduced body cell mass or skeletal muscle mass^8,9^. Both fluid overload and malnutrition have already been reported as major risk factors for morbidity and mortality in HD patients^10–13^. Fluid overload itself may be directly associated with malnutrition, due to the following pathophysiology: fluid overload causes bowel edema, and immune activation can occur due to the translocation of bowel endotoxins into the circulation^14^. This inflammatory response may lead to malnutrition via an increase in protein catabolism and muscle wasting^15,16^. Moreover, fluid overload may increase vessel wall stress, thereby leading to atherosclerosis^17^. Kim et al.^5^ reported that the ECF/ICF ratio might reflect malnutrition, inflammation, and atherosclerosis, which are the core elements of PEW^1^. They also showed that the ECF/ICF ratio is an indicator of new cardiovascular events and all-cause mortality^5^. We have recently demonstrated that the ECF/ICF ratio is independently associated with GNRI, a well-known marker of PEW^4^. Moreover, we have also suggested that the ECF/ICF ratio may be a promising predictor of all-cause and cardiovascular mortality and that the combination of the ECF/ICF ratio and GNRI could increase the predictability of mortality^4^.

The nutritional status can change constantly; therefore, it may have to be assessed repeatedly. We recently reported that the annual change in GNRI improved the predictive accuracy for mortality; we also predicted all-cause and cardiovascular mortality in patients undergoing HD^7^. In this context, although no study has evaluated the relationships between the change in ECF/ICF ratio and clinical outcomes in this population, we speculated that the ∆ECF/ICF ratio could also predict mortality and improve the predictability for mortality.

According to the clinical guideline from the Japanese Society for Dialysis Therapy, the assessment of blood pressure, lower extremity edema, and cardiothoracic ratio are recommended to set dry weight, but there is no description for BIA^18^. Thus, in this study, dry weight was mainly determined by CTR, which can reflect fluid overload. Indeed, the CTR increased in the order of G1 to G4, and an increased CTR was associated with an increased risk of all-cause and cardiovascular mortality. On the other hand, BIA-measured ECF/TBW ratio was used as a reference tool to set dry weight in the daily practice in our clinic. We did not set the specific target value of ECF/TBW ratio, but relative change of ECF/TBW ratio was assessed to set dry weight in individual HD patient. Therefore, these might have naturally affected the results of the ECF/ICF ratio. Thus, in the present study, the ECF/ICF ratio was adjusted by CTR in the Cox analysis, and CTR was also included in the baseline risk model.

On the other hand, some previous studies reported that ECF/TBW ratio was a maker of the fluid volume status and was sometimes used to set dry weight in HD patients^19,20^. Recently, Kim et al.^21^ and Pérez-Morales et al.^22^ have demonstrated that volume overload assessed by BIA-measured ECF/TBW ratio was a predictor of all-cause and cardiovascular mortality. However, Kim et al.^5^ previously reported that ECF/ICF ratio but not ECF/TBW ratio was an independent predictor of all-cause and cardiovascular event. They speculated that ECF/TBW ratio did not reflect changes within the body fluid volume, because changes in the ECF volume was accompanied by simultaneous changes in the ECF components of TBW: an increase of ECW/TBW ratio may be caused by a reduction in TBW because of a loss of ICW. In the present study, although baseline ECF/TBW ratio but not ΔECF/TBW ratio was an independent predictor for mortality, both ECF/ICF ratio and ΔECF/ICF ratio were independent predictors for all-cause and cardiovascular mortality. Therefore, our findings may suggest that longitudinal changes of ECF/ICF ratio may be superior to those of ECF/TBW ratio for evaluating mortality.

In the present study, the ECF/ICF ratio was found to be significantly increased during the first year; however, the ∆ECF/ICF ratio was negatively correlated with the baseline ECF/ICF ratio. In addition, patients with an increased ∆ECF/ICF ratio had a higher risk of mortality than those without an increased ∆ECF/ICF ratio, even in patients with a lower baseline ECF/ICF ratio. These findings may suggest that patients with a good baseline nutritional status but a subsequently deteriorated nutritional status may have increased risks for mortality; therefore, repeated measurements of the ECF/ICF ratio may be useful to stratify the risks for all-cause and cardiovascular mortality in this population.

Regarding model discrimination, the C-index for all-cause mortality significantly improved by adding the ∆ECF/ICF ratio alone to the established risk model, including the GNRI. Moreover, the C-index was maximized when both the baseline ECF/ICF ratio and ∆ECF/ICF ratio were added to the established risk model. In addition, for cardiovascular mortality, the C-index tended to improve, and the NRI and IDI significantly improved with the addition of both the baseline ECF/ICF ratio and ∆ECF/ICF ratio to the established risk model. Therefore, these results may also support the clinical usefulness of regular measurements of the ECF/ICF ratio in predicting all-cause and cardiovascular mortality in HD patients.

Several limitations should be considered in this study. First, this retrospective study included a relatively small number of patients undergoing maintenance HD at a single center. Second, this study included both prevalent HD patients who have already undergone HD before January 2008 and incident HD patients who initiated HD therapy after January 2008. The latter accounted for more than half, therefore HD vintage was relatively short. Thus, our findings might not be simply applicable to all HD patients. Third, the present study included only Japanese HD patients; therefore, our findings might not be generalizable to patients undergoing HD in other countries. Fourth, the associations between the ∆ECF/ICF ratio and mortality were examined in this study; however, the optimal duration of the changes in the ECF/ICF ratio remains unclear. Further prospective large-scale multicenter studies are needed to validate our results.

In conclusion, the ECF/ICF ratio increased in the first year, and an increased ∆ECF/ICF ratio was independently associated with an increased risk of all-cause and cardiovascular mortality in patients undergoing HD. Moreover, the predictive accuracy of mortality improved after the addition of the ∆ECF/ICF ratio to a model with established risk factors, including the GNRI and baseline ECF/ICF ratio. Therefore, it is recommended that the ECF/ICF ratio be serially evaluated to accurately predict mortality in this population.

## Methods

### Study participants

We conducted a retrospective study including 247 patients who underwent maintenance HD for at least 6 months and regularly underwent BIA between January 2008 and December 2019. In our clinic, BIA was performed once a month unless being hospitalized in the entire HD patients. This study was conducted using the medical charts of the outpatient clinic at the Matsunami General Hospital (Kasamatsu, Gifu, Japan) between January 2008 and December 2019. Patient data were fully anonymized prior to access, and the ethics committee of Matsunami General Hospital waived the requirement for informed consent. This study adhered to the principles of the Declaration of Helsinki, and the study protocol was approved by the ethics committee of Matsunami General Hospital (No. 482).

### Data collection

The following data were collected from medical charts: age, sex, dialysis vintage, history of hypertension, diabetes, cardiovascular disease (CVD), dry weight, and height. Hypertension was defined as systolic blood pressure ≥ 140 mmHg and/or diastolic blood pressure ≥ 90 mmHg before the HD session or antihypertensive drug usage. Diabetes was defined as a history of anti-diabetic therapy. In this study, CVD was defined as angina pectoris, myocardial infarction, cerebral apoplexy, and heart failure. Blood samples were collected with the patient in a supine position before the HD session, which was conducted on a Monday or Tuesday. BIA was performed on the same day, shortly after the HD session. Body composition was evaluated using a multi-frequency (2.5–350 kHz) body composition analyzer (MLT-550N, SK Medical, Japan), using the wrist-ankle method. ECF and ICF were obtained, and the ECF/ICF ratio was calculated. The ECF/ICF ratio was calculated at baseline and after one year, and the ΔECF/ICF ratio was calculated as follows: ΔECF/ICF ratio (%) = (ECF/ICF ratio after one year − baseline ECF/ICF ratio)/baseline ECF/ICF ratio × 100.

### Follow-up study

The primary endpoint was all-cause mortality, and the secondary endpoint was CVD mortality. Patients were divided according to the median baseline ECF/ICF ratio. Patients in whom the ECF/ICF ratio could not be assessed after the first year were excluded and the remaining patients were divided into two groups based on the increased or decreased ECF/ICF ratio during the first year. Thereafter, patients were divided into four subgroups based on the combination of the median baseline ECF/ICF ratio and the increased or decreased ECF/ICF ratio during the first year (ΔECF/ICF ratio < 0% vs. ≥ 0%): Group 1 (G1), ECF/ICF ratio < 0.563 and ΔECF/ICF ratio < 0%; G2, ECF/ICF ratio ≥ 0.563 and ΔECF/ICF ratio < 0%; G3, ECF/ICF ratio < 0.563 and ΔECF/ICF ratio ≥ 0%; G4, ECF/ICF ≥ 0.563 and ΔECF/ICF ≥ 0%. In the present study, the follow-up period was defined as the interval from the date when BIA was performed to the date of death or the date when moving out to another hemodialysis unit. Patients who were alive were censored in December 2020.

### Statistical analyses

Normally distributed variables are expressed as the means ± standard deviations, and non-normally distributed variables are expressed as medians and interquartile ranges. Differences among the four subgroups divided by the median ECF/ICF ratio and the increased or decreased ECF/ICF ratio during the first follow-up year were compared by one-way analysis of variance or the Kruskal–Wallis tests for continuous variables or the chi-squared test for categorical variables. The association between ΔECF/ICF ratio and baseline ECF/ICF ratio was evaluated using Spearman’s rank correlation coefficient. The Kaplan–Meier method was used to estimate the survival rate, and the log-rank test was used to analyze the differences. Univariate Cox proportional hazard regression analysis was used to estimate HRs and 95% CIs for all-cause and cardiovascular mortality. The multivariate regression model included all significant covariates in the univariate analysis and sex. Cox analysis for baseline ECF/ICF ratio was performed in the entire group of HD patients (N = 247), whereas that for ΔECF/ICF ratio and four subgroup (G1 to G4) was performed in the remaining HD patients (N = 198) who excluded during the first year of follow-up.

To examine whether the accuracy for predicting mortality can improve after the addition of the baseline ECF/ICF ratio and/or ΔECF/ICF ratio to the baseline model including sex and variables which were significant in the univariate Cox analysis, we calculated the C-index, NRI, and IDI. The C-index was defined as the area under the receiver operating characteristic curve between individual predictive probabilities for mortality and the incidence of mortality, and it was compared between the baseline model and the adjusted model with the baseline ECF/ICF ratio and/or the ΔECF/ICF ratio^23^. The NRI was used to show a relative improvement in the number of patients for whom the predicted mortality risk improved, and the IDI was used as an indicator of the average improvement in the predicted mortality risk after adding the new variables to the baseline model^24^.

All statistical analyses were performed using IBM SPSS version 21 (IBM Corp., Armonk, NY, USA). A P-value < 0.05 was considered statistically significant.

### Ethical approval

This study adhered to the principles of the Declaration of Helsinki, and the study protocol was approved by the ethics committee of Matsunami General Hospital (No. 482). The requirement for informed consent was waived because patient data were anonymized.
